# Fight, Flight, – Or Grab a Bite! Trait Emotional and Restrained Eating Style Predicts Food Cue Responding Under Negative Emotions

**DOI:** 10.3389/fnbeh.2020.00091

**Published:** 2020-06-03

**Authors:** Rebekka Schnepper, Claudio Georgii, Katharina Eichin, Ann-Kathrin Arend, Frank H. Wilhelm, Claus Vögele, Annika P. C. Lutz, Zoé van Dyck, Jens Blechert

**Affiliations:** ^1^Department of Psychology, Division of Health Psychology, Paris-Lodron-University of Salzburg, Salzburg, Austria; ^2^Department of Psychology, Division of Clinical Psychology and Psychopathology, Paris-Lodron-University of Salzburg, Salzburg, Austria; ^3^Department of Behavioural and Cognitive Sciences, Institute for Health and Behaviour, University of Luxembourg, Esch-sur-Alzette, Luxembourg

**Keywords:** emotional eating, restrained eating, mood induction, food cue reactivity, multilevel modeling, P300, corrugator supercilii

## Abstract

In today’s society, obesity rates are rising as food intake is no longer only a response to physiological hunger signals that ensure survival. Eating can represent a reward, a response to boredom, or stress reduction and emotion regulation. While most people decrease food intake in response to stress or negative emotions, some do the opposite. Yet, it is unclear who shows emotional overeating under which circumstances. Emotion regulation theories describe emotional overeating as a learned strategy to down-regulate negative emotions. Cognitive theories, by contrast, attribute emotional overeating to perceived diet breaches in individuals who chronically attempt to diet. After consuming “forbidden foods”, they eat more than individuals who do not restrict their food intake. This laboratory study investigated emotional overeating by exposing individuals to a personalized emotion induction while showing images of palatable foods. Outcome variables indexed cue reactivity to food images through picture ratings (valence, desire to eat), facial expressions (electromyography of the corrugator supercilii muscle), and brain reactivity by detecting event-related potentials (ERPs) by means of electroencephalography (EEG). The influence of emotion condition (negative, neutral) and individual differences (self-reported trait emotional and restrained eating) on outcome variables was assessed. Valence ratings and appetitive reactions of the corrugator muscle to food pictures showed a relative increase in the negative condition for individuals with higher emotional eating scores, with the opposite pattern in lower scores. Desire to eat ratings showed a similar pattern in individuals who showed a strong response to the emotion induction manipulation, indicative of a dose-response relationship. Although no differences between conditions were found for ratings or corrugator activity with restrained eating as a predictor, an ERP at P300 showed increased activation when viewing food compared to objects in the negative condition. Findings support emotion regulation theories: Emotional eaters showed an appetitive reaction in rating patterns and corrugator activity. EEG findings (increased P300) suggest a motivated attention toward food in restrained eaters, which supports cognitive theories. However, this did not translate to other variables, which might demonstrate successful restraint. Future studies may follow up on these findings by investigating eating disorders with emotion regulation difficulties.

## Introduction

While food search and metabolic energy balance represented a key challenge for the longest part of human evolution, today’s modern, affluent societies secure the continued supply with energy dense, palatable and affordable foods. Eating has since assumed multiple roles and functions beyond energy homeostasis: it serves enjoyment, relief from boredom and sometimes, stress reduction and emotion regulation. Although such non-homeostatic roles of eating are natural, emotional eating is involved in difficulties with losing weight, linking it with the alarming rates of overweight and obesity worldwide (World Health Organization [WHO], 2018). Emotional eating has also been identified as a risk factor for developing binge eating and associated eating disorders as early as in middle school (Pearson et al., 2012). In the past three decades, these significant health concerns, along with interest in the basic science of emotions and eating behaviors motivated intense research into the connections between emotions and eating (Vögele et al., 2018). Different theories have attempted to explain emotional eating, including physiological theories on stress reactions, as well as psychological theories that consider individual traits.

From a physiological, evolutionary point of view, intense stress and negative emotions are expected to trigger a fight-or-flight response involving the sympathetic nervous system, resulting in increased alertness and energy provision from bodily stores and decreased appetite and food intake (Torres and Nowson, 2007). Yet, food cravings with subsequent overeating in response to stress can also be observed (Yau and Potenza, 2013) leading to the question what other processes override this basic, defensive mechanism. While physical stressors have become infrequent in the last centuries, emotional, chronic stressors are more present in daily life and have been linked to activation of the hypothalamic–pituitary–adrenal (HPA) axis. According to physiological theories, excessive “comfort food” consumption in response to emotional stress of this sort could be related to glucocorticoids (Bazhan and Zelena, 2013). Consequences of HPA axis dysregulation, e.g., excessive glucose release from the liver, have been shown to contribute to the development of unhealthy eating habits and obesity because of chronic environmental stress (Bose et al., 2009), albeit mechanisms are still unclear. Physiological theories predict that stronger responses to a stressor should alternate appetitive responding more (Vallès et al., 2000), possibly due to different stress axis activation and metabolic reaction.

Accompanying physiological adaptations to negative emotions and stress, several psychological theories and mechanisms of emotional overeating have been proposed. As early as 1955, Hilde Bruch ascribed emotional overeating to early breast-feeding experiences, conflating sensations of hunger relief with emotional caring. Booth (1994) proposed a learning model that predicts positive emotional effects of eating to counter negative emotions, thereby negatively reinforcing emotional overeating. Thus, trait emotional eating might serve as an emotion regulation strategy. Yet, there are rivaling theories, most prominently cognitive theories based on disinhibition of restraint. Research in the domain of trait restrained eating has shown that some individuals who chronically diet form relatively rigid diet rules. When violating such a diet rule, for example by having to consume high-calorie, “forbidden” foods in an experiment prior to a taste test, most restrained eaters disregard their diet rules on the taste test and thus disinhibit their suppressed eating desires (Herman and Mack, 1975). Crucially, emotions can disinhibit rigid restraint rules in restrained eaters in a similar fashion: Multiple studies have shown that restrained eaters do not only eat more in response to a “diet violation,” but also when experiencing negative emotions, possibly because these emotions use up the cognitive resources needed for adhering to diet rules (Cardi et al., 2015). Taken together, on the one hand, learning theories suggest that emotional overeating primarily yields an emotional regulatory function – and individuals overeat to regulate negative emotions; on the other hand, cognitive theories suggest that emotional overeating does not necessarily have an emotional regulatory function but results from disinhibition in restrained eaters.

Departing from this theoretical debate, evidence for emotional overeating is surprisingly inconsistent and current debates revolve around several points that require clarification. First, recent reviews have yielded inconsistent results as of which theory best explains emotional overeating. Whereas Cardi et al. (2015) found evidence for emotional overeating in negative mood across studies and even more pronounced in restrained eaters and individuals with binge eating symptomatology, a more comprehensive and recent meta-analysis questioned that. Evers et al. (2018) showed emotional overeating only in trait restrained and not in trait emotional eating. Nevertheless, the authors note large variability in methodology and analytic strategies across studies and stress the importance of the method of emotion induction. Since trait emotional and restrained eating are operationalized with questionnaires, a cut-off debate of only including extreme scorers evolved (Van Strien et al., 2012) however, this contradicts dimensional models of psychopathology and personality (Cuthbert and Insel, 2013). Therefore, the current study applied a state of the art multilevel modeling (MLM) approach based on continuous trait emotional and restrained eating scores.

The present study investigates to what extent traits (emotional or restrained eating) and emotional states (negative compared to neutral emotions as well as individual strength of emotion reactivity) predict changes in appetitive responding to food images on experiential, autonomic, facial and neural outcome measures. Participants completed a previously validated experimental rating task (Blechert et al., 2014) consisting of food ratings in a neutral compared to a negative condition, which allowed for a high level of control over error variances. Preceding this task, negative emotions were induced with a validated procedure based on idiosyncratic autobiographic recall (Hilbert et al., 2011), this also ensured that emotional changes would cause changes in appetitive responding (and not the reverse, Adriaanse et al., 2011b). High density EEG was recorded, covering all relevant neural regions to reveal underlying mechanisms. As an indicator for a physiological appetitive reaction, corrugator supercilii activity was measured, a muscle that reacts with relaxation to positive stimuli and with contraction to negative stimuli (Larsen et al., 2003). As an indicator for general physiological arousal, heart rate (HR) and heart rate variability (HRV) data during the two conditions (neutral, negative) was collected. By including a sufficiently large sample with enough orthogonal variation on trait emotional and restrained eating, the present study was set up to model the relative predictive power of each of these traits on emotional overeating.

**FIGURE 1:**
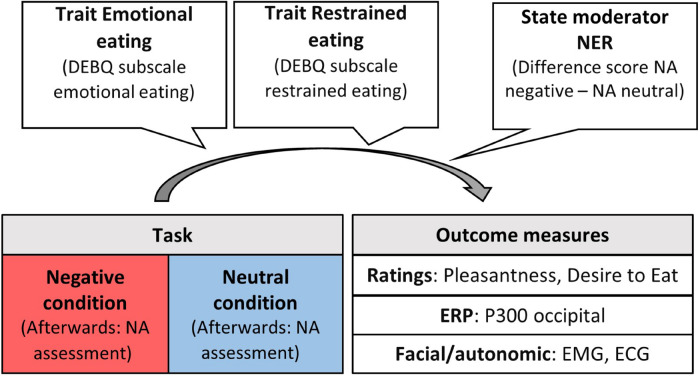
Moderators, task conditions, and outcome measures for appetitive responding. NER, negative emotional reactivity; NA, negative affect; DEBQ, dutch eating behavior questionnaire; ERP, event-related potential; EMG, electromyography; ECG, electrocardiogram.

Measuring experiential, autonomic, facial and neural responses to induced negative emotions simultaneously allowed to get an overall picture for processes that lead to a motivational readiness to engage in emotional overeating. Under this approach, we addressed the following research questions. Based on (animal) literature suggesting appetite suppression due to activation of the sympathetic nervous system when experiencing stress (Vallès et al., 2000), we expected lower pleasantness and desire to eat rating scores for food pictures under negative emotion in individuals without a tendency for emotional overeating (that is, either low scores on emotional or restrained eating). This stress reaction should also be evident in increased facial frowning detected by the corrugator muscle. Individuals prone to emotional overeating should show opposite rating and corrugator reactivity patterns. Emotion regulation theories predict an emotional overeating effect in individuals with high emotional eating scores. Further, this might show in cortical activity in parietal-occipital, attentional areas, as indexed by the P300, particularly in emotional eaters as in Blechert et al. (2014). In contrast, disinhibition of restraint theories predict this effect only individuals with higher trait restrained eating. In addition, this elevated reactivity could be accompanied by decreased frontal brain activity due to the disinhibiting effects of negative emotions on regions that exert cognitive control over cravings (Kober et al., 2010; Blechert et al., 2014; Wood et al., 2016). Last, based on physiological theories, we investigated to what extent the relationship between appetitive responding and eating traits (emotional vs restrained) is modulated by individual differences in the strength of emotional reactivity (more suppression vs more emotional overeating). See Figure 1 for a visualization of these research questions.

## Materials and Methods

### Participants

Eighty female, right-handed participants aged between 16 and 50 years (*M* = 21.9, SD = 3.77) with normal average BMI (*M* = 22.3, SD = 3.09) were recruited in introductory psychology classes at the University of Salzburg, Austria, via newspapers and web portals. An all-female sample was chosen because women have a higher risk of eating disorders (Galmiche et al., 2019) and report eating more often as a coping mechanism when experiencing negative emotions or stress (Greeno and Wing, 1994). Further, the association between craving and eating pathology is stronger in women (Chao et al., 2016) and succumbing to cravings is more frequently related to negative feelings in women (Lafay et al., 2001). Sample size was based on Blechert et al. (2014), *N* = 45, and the additional consideration to include enough high and low restrained eaters. Emotional (*M* = 2.51, SD = 0.76) and restrained eating (*M* = 2.48, SD = 0.81) were known to correlate only weakly in the current sample (*r* = 0.120, *p* = 0.290, see scatterplot in Supplementary Information A), allowing independent, orthogonal modeling. Self-reported exclusion criteria were vegetarianism, food allergies, diabetes, and other disorders that affect digestion and eating behavior; a history of eating disorders; current substance abuse; and neurological disorders. One participant was excluded due to current substance abuse (final sample: *n* = 79). Due to poor data quality, six participants were excluded from EMG analyses (final sample: *n* = 73) and 10 from EEG analyses (final sample: *n* = 69). For participating in the lab part of the study, participants received either study credits or 30€ as compensation.

### Procedure

#### Inclusion and Exclusion

Participants that met inclusion criteria first underwent a modified structural clinical interview for DSM-IV (SCID I; Wittchen et al., 1997) to assess current and lifetime mental disorders and current eating pathology according to criteria of the Diagnostic and Statistical Manual of Mental Disorders 5 (DSM-5; American Psychiatric Association, 2013). They further installed a smartphone app on their phone for a seven-day ecological momentary assessment (EMA) prior to the laboratory experiment (data reported elsewhere), and received a link to complete trait questionnaires and demographic information online during the EMA phase.

#### General Procedure and Idiosyncratic Emotional Script Generation

The laboratory session was scheduled after completing the EMA phase, always at 3 pm to avoid circadian variation. To limit variation in hunger, participants were instructed to consume one of five preset lunch options at noon, each containing ∼550 kcal. At the beginning of the testing session, compliance with these instructions was assessed verbally and hunger, appetite, and fullness were in the medium range for all participants on a scale from 1 = “not at all” to 9 = “very much” (*M* = 4.71, SD = 1.35). Laboratory testing started with informed consent (for *n* = 3 underage participants, parents’ consent was obtained) and initial questionnaires, including negative affect at baseline. Further, recent situations were discussed, which had left the participant experiencing negative emotions like sadness or frustration (excluding traumatic events). Participants estimated how well they remembered the situations, how negative the situations were, and how much distress they felt when recalling the situation. The situation with the highest scores on all variables was chosen and further explored regarding what had happened, when, where, with whom, and which thoughts, emotions, physical sensations, behaviors, and consequences occurred. With this information, the experimenter then generated a script of eight sentences. These were then presented to the participant during the emotional eating task to induce negative emotions. Further, a script for the neutral condition in the emotional eating task was generated. Piloting showed that it was rather difficult for participants to recall a truly neutral situation, thus they chose one out of two pre-scripted situations (either brushing their teeth or going to work/school). These neutral scripts were of equal length as the negative scripts and participants were given the opportunity to adjust the sentences so that they best matched their memory of the situation. After this preparatory work for the emotional eating task, physiological sensors were attached (i.e., EEG, respiration, EMG, ECG), a rating of current hunger was taken, and an interoception task (∼10 min) (results reported elsewhere) completed.

#### Emotional Eating Task

**FIGURE 2:**
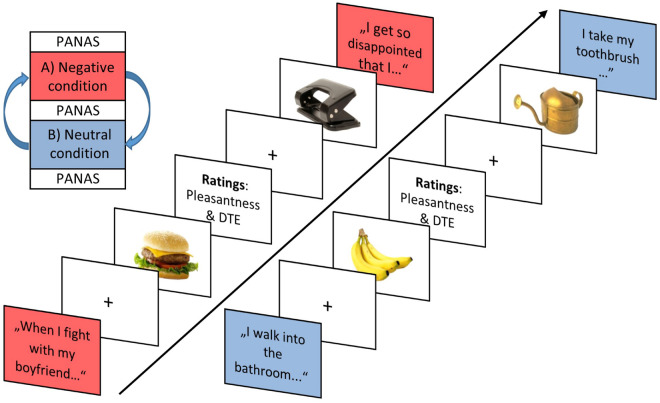
Trial sequence exemplifying two idiosyncratic scripts of the negative and neutral condition with interleaved food/object image presentations alongside respective ratings in the negative condition (red box) and the neutral condition (blue box). The 52 different pictures were divided up evenly among the eight different sentences, resulting in six to seven picture presentations after a sentence presentation. DTE, desire to eat.

The ∼40–50 min task comprised a neutral and negative condition, which were presented in alternating order across participants (Figure 2) enclosed by PANAS ratings as manipulation check. The eight scripted idiosyncratic sentences were first read out aloud by the experimenter prior to each condition. During each condition block, then, sentences were presented in written form on screen, interleaved with the presentation of food and object pictures (and picture rating prompts). Each of the eight negative and neutral sentences was repeated once within each condition block. Also, each of the 26 food and 26 object pictures were presented twice per condition, resulting in 104 image presentations per condition and a total of 208 image presentations for the study. In addition, each image was rated once per block for pleasantness (all images; 104 ratings) and current desire to eat (food images only; 52 ratings) on a visual analog scale ranging from 0 (“very unpleasant”/“no desire to eat”) to 100 (“very pleasant”/“strong desire to eat”). Ratings were displayed either after the first or second picture presentation. A food choice task (Georgii et al., 2019) followed the emotional eating task. The University’s ethics committee approved the study design; relevant guidelines and regulations were met during all experiments.

### Measures

#### Dutch Eating Behavior Questionnaire (DEBQ German (FEV-I); 33 Items; Grunert, 1989)

The DEBQ is a 33-item measure consisting of three subscales. In this study, means of the subscales for trait emotional eating (13 items; e.g., “Do you have the desire to eat when you are irritated?”), and trait restrained eating (10 items; e.g., “Do you try to eat less than you would like to eat at mealtimes?”) were included.

#### Positive and Negative Affective Schedule (PANAS State German; Krohne et al., 1996)

At the beginning of the experiment, after the negative condition, and after the neutral condition participants filled out the PANAS as an emotion induction check. It consists of 20 adjectives, half of them inquiring positive and the other half inquiring negative affective states. Participants answer how much they experience a certain affective state described by an adjective on a 5 point Likert scale ranging from 1 = “just a little/not at all” to 5 = “very much”. Analyses in this study focus on the negative affect (NA) subscale as a construct of interest in the context of emotional eating. PANAS scores assessed after the neutral emotion condition were subtracted from the scores taken after the negative emotion condition to create the “emotional reactivity score”.

#### Electroencephalography (EEG)

Autonomic, facial, and neural measures were recorded with a 64-channel-amplifier (TMSi, Twente Medical Systems International, EJ Oldenzaal, Netherlands) at a sampling rate of 512 Hz. Online average reference EEG was recorded from 63 actively shielded, equidistant, and passive electrodes (sintered Ag/AgCl electrodes) and a ground electrode attached to the left wrist. Impedances were kept below 10 kΩ. Preprocessing in EEG lab (Delorme and Makeig, 2004) involved down-sampling (256 Hz), low-pass filtering (70 Hz), high-pass filtering (0.1 Hz), and notch filtering (45–55 Hz). Noisy channels were identified through visual inspection and removed, data was re-referenced to average and eye blinks and –movements were removed in an independent component analysis (ICA) using AMICA (Palmer, 2016). Then, 1500ms pre-stimulus to 2400 ms post-stimulus epochs were extracted unless large artifacts were evident upon visual inspection. Lastly, previously removed channels were interpolated.

Regions of interest were selected based on visual inspection of topography plots and difference waves (Figure 5). Other than in the previous report (Blechert et al., 2014), a P300 was evident between 200–400 ms at a parieto-occipital electrode cluster (5LB, 5LC, 7L, 8L, 9L, 7Z, 8Z, 9Z, 7R, 8R, 9R, 5RB, 5RC). Mean amplitudes were calculated and the ERP was indexed relative to a 300ms pre-stimulus baseline.

#### Corrugator Electromyography (EMG)

To record facial EMG activity, two 2 mm inner diameter Ag/AgCl electrodes were placed on the corrugator supercilii muscle region above the left eye, following established guidelines (Fridlund and Cacioppo, 1986). After 28 Hz high-pass filtering (removal of movement artifacts) and 50 Hz notch filtering, rectification and smoothing (50 ms moving average) was applied. After preprocessing, noisy signal segments were manually rejected through visual inspection. For visual inspection on picture level analyses, mean amplitudes of five 500 ms time segments, spanning 2500 ms picture presentation time were extracted before subtracting mean activation during a 500 ms pre-stimulus baseline.

#### Electrocardiogram (ECG)

Generalized emotional or arousal related effects of the conditions might also be evidenced in cardiac activity. Thus, ECG was recorded using disposable solid-gel snap electrodes placed on the upper sternum and distal end of the left costal arch. Data were inspected offline and artifacts were corrected manually with ANSLAB 2.6 (Blechert et al., 2016). The number of heartbeats in each condition was automatically determined by detecting the number of R-spikes. Overall HR and HRV scores (natural logarithm of fast-Fourier transformation derived HRV for very low, low, and high frequency band power) for each condition were calculated. No picture level ECG responses were calculated due to the fast picture presentation mode and the resulting inability of the ECG to return to baseline. Furthermore, emotional reactivity can also be indexed on HR and HRV, similar to negative PANAS, by subtracting means of the neutral condition from means of the negative condition.

#### Multilevel Modeling

Statistical models that regressed food image reactivity on condition (negative, neutral) and eating traits (emotional eating, restrained eating) as well as individual differences in emotional reactivity (defined by a difference score of induced negative emotions) were built (see Figure 1). Person-level predictors (eating traits, emotional reactivity) were grand mean centered. To control for general mood effects on rating tendencies, mean pleasantness ratings of objects pictures were subtracted from mean pleasantness ratings of food pictures separately for each condition. These two difference scores were then submitted to an MLM with condition as a random intercept, testing for food specific effects. The same approach was used for autonomic, facial and neural measures.

Desire to eat ratings were treated differently: Since they were available for food images only, no difference score with object ratings could be calculated. This allowed to model ratings on single trial level without calculating a mean score for each condition. To test whether this more complex model would be a better fit to analyze data, intra-class correlation coefficients (ICCs) were calculated in a first step. Significant ICCs indicate that desire to eat as a dependent variable is nested within the crossed random predictors Condition, Participant and Picture Number (Bliese, 2000). This was the case for all predictors, all ps < 0.001, therefore a cross-classified, nested structure could be assumed. Thus, an MLM with desire to eat modeled on the single trial level (not averaged across trials) and both nested within Condition (Level-2) and within Participant (Level-2; crossed structure) was chosen as the best fitting model.

MLMs were analyzed in RStudio (Allaire, 2012) using lme4 (Bates et al., 2015) for linear mixed-effects models with crossed random effects structure (Bliese, 2013). Results were plotted using ggplot2 (Wickham, 2016). A hypothesis-driven, two-step, backward modeling approach was chosen. In a first step, all theoretically important predictors were included in the MLM (eating trait, emotional reactivity, Condition, and all their two- and three-way interactions) alongside covariates of no interest (condition order, i.e., whether the neutral or the negative condition came first, negative emotions at baseline, individual food picture, and trial position within a condition for effects of habituation). In a second step, models were reduced by stepwise exclusion of predictors that were not significant for each outcome variable. Akaike Information Criterion (AIC) served as an indicator of model fit, with lower AICs of the final model indicating a better fit. A random slope for participants was added, allowing the strength of relation between predictors and dependent variable to vary between participants.

## Results

### Idiosyncratic Script Content Analysis

Idiosyncratic scripts were classified independently by two raters involved in the study. Most of the negative situations contained conflicts with other family members (25%), followed by friends (22%) and partners (16%). Fifteen percent were other interpersonal situations, 20% were various non-social situations. 42% of the situations mainly invoked anger, 34% induced sadness and 24% anxiety. As a neutral situation, 65% of participants chose brushing their teeth, the rest chose going to work/school by various means of transport. Participants indicated that they could visualize the scenarios well (*M* = 7.49, SD = 1.12, on scale from 1 = “not at all” to 9 = “very much”).

### Manipulation Check

**FIGURE 3:**
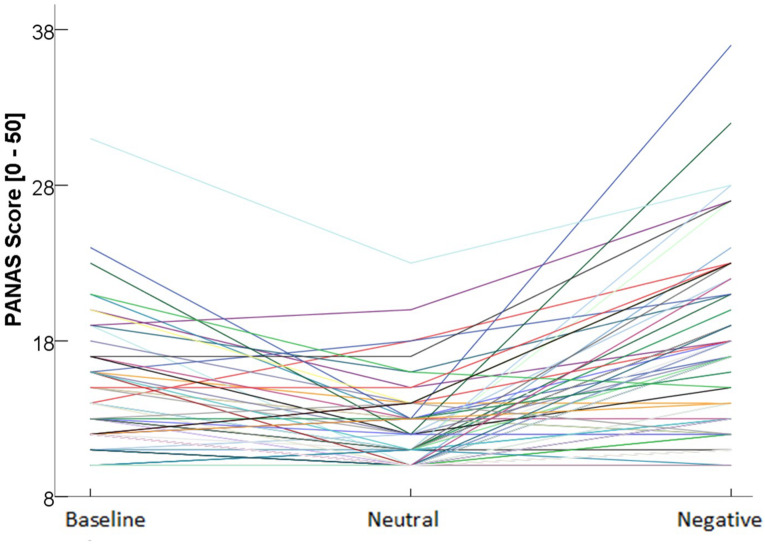
Individual negative affect scores at different time points.

Changes in NA scores (PANAS) confirmed effective emotion induction: the Condition (after negative-baseline, after neutral-baseline) × Order (negative first, neutral first) ANOVA with repeated measures on Condition revealed a main effect for Time, *F*(2,77) = 44.6, *p* < 0.001, ηp^2^ = 0.367 and no interaction effect with Order, *F*(2,77) = 0.417, *p* = 0.598, ηp^2^ = 0.005. Successful emotion induction was evident in more negative PANAS ratings after the negative, compared to the neutral condition, mean difference MD = −4.71, *p* < 0.001, 95% CI [−6.36, −3.06]. Relative to baseline, measured well before emotion induction, the neutral condition elicited less NA, MD = 1.75, *p* = 0.034, 95% CI [0.098, 3.40], and the negative condition elicited more NA, MD = –2.96, *p* < 0.001, 95% CI [−4.61, −1.31]. Trait emotional eating was not associated with altered negative affect at baseline, *r* = 0.174, *p* = 0.124; however, individuals with higher restrained eating reported slightly more negative affect at baseline, *r* = 0.228, *p* = 0.044; therefore, baseline negative affect was added as covariate of no interest in all analyses (fixed main effect, no interactions). No effects on the dependent variables were obtained when using a “physiological emotional reactivity score” (difference score of mean EMG or ECG activity for each condition) in all analyses. Figure 3 shows PANAS scores for the three measurement points and illustrates the large individual variability in negative affect after the negative condition, which highlights the importance of including the (subjective) emotional reactivity variable in all analyses.

### Food Picture Pleasantness Ratings

**FIGURE 4:**
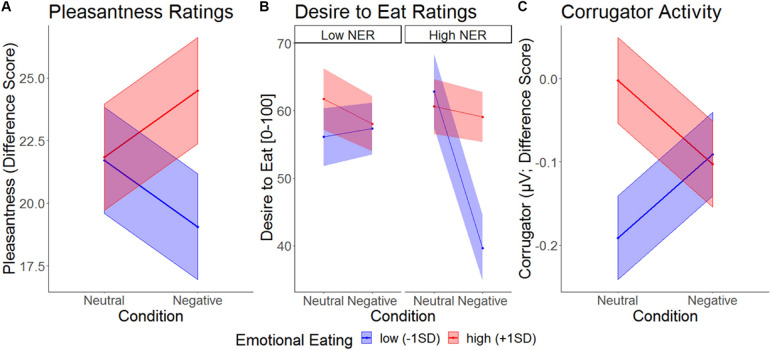
Picture responses with simple slopes illustrating different patterns in the two conditions between high and low emotional eaters for **(A)**, pleasantness ratings (difference score Food – Objects), **(B)**, desire to eat ratings (food only), and **(C)**, activity of corrugator supercilii (difference score Food – Objects). NER, negative emotional reactivity.

Modeling the effect of Condition, eating trait, and emotional reactivity on pleasantness ratings (for foods relative to objects) revealed a trend main effect of trait emotional eating that was moderated by Condition (Table 2). As can be seen in Figure 4, high emotional eaters rated food pleasantness higher than low emotional eaters specifically in the negative condition. The same model with trait restrained instead of emotional eating did not reveal main effects of restrained eating, an interaction with emotional reactivity, but no interaction with Condition (Supplementary Information B).

### Food Picture Desire to Eat Ratings

A Condition main effect on desire to eat (only obtained for food images) suggesting appetite suppression should be interpreted in the context of three two-way and the three-way interaction (Table 1). Inspection of Figure 4 suggests a similar Condition × Emotional Eating two-way interaction as for pleasantness, with the clearest picture, however, emerging in individuals high in emotional reactivity (3-way interaction): among high-reacting individuals, low emotional eaters showed extreme appetite suppression under negative emotions, while this emotional suppression was attenuated in high emotional eaters. In other words, among strong responders, trait restrained eating went along with a relative increase in appetitive picture responding, our proxy measure for emotional overeating. By contrast, when investigating trait restrained instead of emotional eating in this model, we found no main effects or interactions (Supplementary Information C).

**TABLE 1:** Full and reduced Multilevel-Models for regression coefficients (*b*) of desire to eat rating for food only, modeled on trial level with trait emotional eating as a predictor (number of observations: *N* = 4108).

	Full model DTE	Reduced model DTE

Fixed effects	*b* (SE)	*b* (SE)
(Intercept)	51.77(3.31)***	53.5(2.57)***
**Predictors**		
Condition	7.04(1.66)***	6.79(1.65)***
Trial	0.03 (0.02)	
Condition order	0.12 (3.46)	
NA baseline	−0.21(0.44)	
NER	−0.80(0.36)*	−0.83(0.36)*
tEE	7.00(2.43)**	6.62(2.33)**
**Interactions**		
Condition × NER	1.11(0.34)**	1.11(0.34)**
Condition × tEE	−6.00(2.23)**	−5.52(2.19)*
NER × tEE	1.26(0.54)*	1.22(0.53)*
Condition × NER × tEE	−1.78(0.50)***	−1.73(0.50)***

**Random effects**	**Variance (*SD*)**	**Variance (*SD*)**

Participant (Intercept)	198 (14.1)	196 (14.0)
Participant | Condition	135 (11.6)	133 (11.5)
Picture number (Intercept)	91.9 (9.58)	91.9 (9.59)
Residual	927 (30.4)	927 (30.4)
AIC	40094	40090
χ^2^	0.592	

DTE, desire to eat; NER, negative emotional reactivity; tEE, trait emotional eating; SE, standard error. Adding a random slope for participant significantly improved DTE predictions (p < 0.001). Significance codes: “***” = p < 0.001; “**” = p < 0.01; “*” = p < 0.05.

### Electroencephalography (EEG)

**FIGURE 5:**
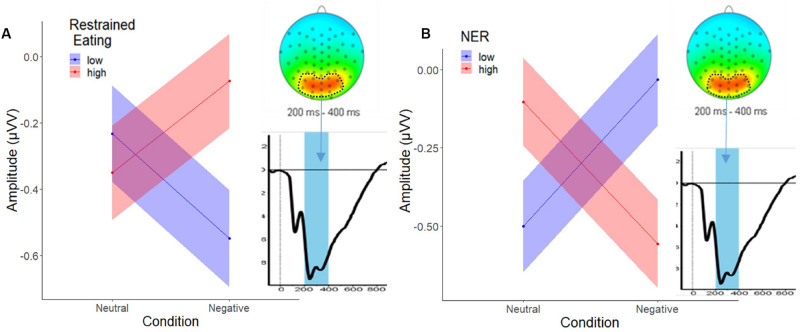
Two-way interaction of **(A)**, restrained eating (±1SD) and condition, and **(B)**, NER (±1SD) and condition at P300. The circled area in the head plot shows the extracted cluster, the blue bar in the waveform graph illustrates the P300 peak observed in the 200–400 ms time window.

The analysis of the parieto-occipital P300 activity (foods minus objects) showed no significant main effects or interactions as a function of trait emotional eating. The model with trait restrained eating as a predictor, however, revealed main effects for restrained eating and emotional reactivity, each interacting with Condition, but no three-way interaction. Inspection of the means in Figure 5 suggest that high-reacting and low restrained individuals showed increased P300 amplitudes to food images (relative to object images) under negative (relative to neutral) emotion whereas their respective counterparts showed decreased food-specific P300 amplitudes under negative emotion. See Supplementary Information D for detailed predictor values.

A right frontal effect in the LPP time range (as in Blechert et al., 2014) was not visible upon inspection of difference waves. To test for potential higher order interactions, we extracted a broad bilateral frontal ERP cluster in the LPP time range (1LB, 1RB, 2LB, 2RB, 2L, 2R, 1Z, 2Z) and a right-frontal cluster (1R, 1RB, 2RB, 2RC, 1RD, as in previous report) between 300–600 ms. See Supplementary Information E for findings on frontal effects.

### Corrugator Electromyography (EMG)

**FIGURE 6:**
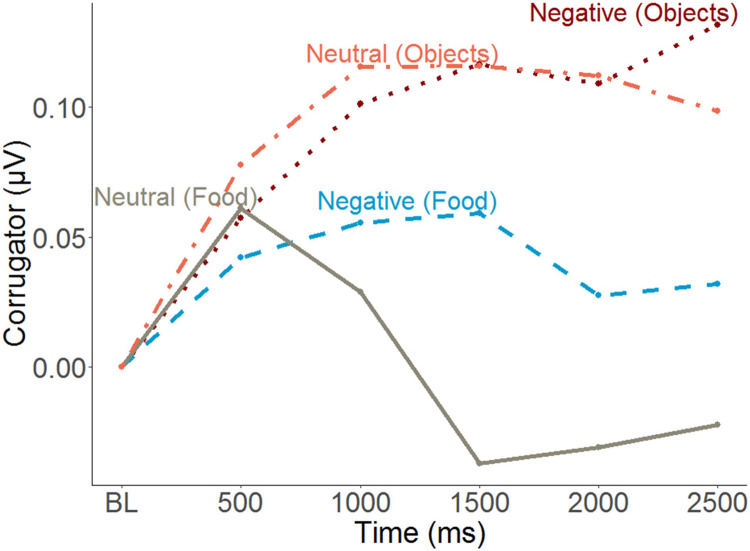
Time course of the corrugator supercilii muscle responses to images as a function of conditions and picture type (mean amplitude of 500 ms segments). BL, Baseline.

The EMG time course suggests a generalized increase in corrugator response within the first 500ms and a differentiated pattern thereafter, with food pictures inducing a decrease relative to objects, and condition effects mainly for foods. To capture this latter phase, averages over the segments covering activity between 500 and 2500 ms were calculated (Figure 6). Furthermore, to capture food specific effects only (and similar to pleasantness and ERP response scoring), object responses were subtracted from food responses prior to statistical analysis. An MLM predicting food-specific corrugator activity yielded main effects of Condition, Emotional Eating and their interaction (Figure 4 and Table 2). Inspection of the slopes in Figure 4 revealed a similar pattern as for pleasantness ratings: in low emotional eaters, corrugator activity increased from the neutral to the negative condition, indexing the negative affective state (or – by analogy – appetite suppression). This pattern reversed in high emotional eaters: corrugator attenuation – potentially indicative of positive and appetitive states – was seen in the negative relative to the neutral condition (Figure 4 and Table 2). Again, there were no main effects or interactions of restrained instead of emotional eating, ps > 0.168. See Supplementary Information C for trait restrained eating results.

**TABLE 2:** Full and reduced Multilevel-Models for regression coefficients (*b*) of Pleasantness and Corrugator Electromyography for a food-object difference score with trait emotional eating as a predictor.

	Full model pleasantness	Reduced model pleasantness	Full model corrugator	Reduced model corrugator

Fixed Effects	*b* (SE)	*b* (SE)	*b* (SE)	*b* (SE)
(Intercept)	21.7(2.03)***	21.8(1.43)***	−0.15(0.05)***	−0.10(0.03)**
**Predictors**				
Condition	1.51 (1.26)		0.07 (0.05)	
Condition order	−1.55(2.99)		0.05 (0.07)	
NA baseline	−0.43(0.39)		0.01 (0.01)	
NER	−0.48(0.33)		0.005 (0.008)	
tEE	4.90(2.20)*	3.57(2.05)^(*)^	0.11 (0.06)	0.12(0.05)*
**Interactions**				
Condition × NER	−4.21(01.66)		−0.003(0.01)	
Condition × tEE	0.32(0.26)*	−3.48(1.63)*	0.14(0.07)*	−0.13(0.06)*
NER × tEE	−0.24(0.49)		0.008 (0.01)	
Condition × NER × tEE	−0.59(0.38)		−0.02(0.01)	

**Random Effects**	**Variance (*SD*)**	**Variance (*SD*)**	**Variance (*SD*)**	**Variance (*SD*)**

Participant (Intercept)	133 (11.6)	132 (11.5)	0.03 (0.17)	0.03 (0.17)
Residual	59.3 (7.70)	60.0 (7.75)	0.09 (0.30)	0.09 (0.30)
AIC	1242	1236	110	103
χ^2^	0.363		0.504	

NER, negative emotional reactivity; tEE, trait emotional eating; SE, standard error. Significance codes: “***” = p < 001; “**” = p < 0.01; “*” = p < 0.05; “^(^*^)^” = p < 0.1.

### Electrocardiogram (ECG)

Comparing HR and HRV during the neutral and negative condition revealed no significant main effects and no interaction with eating trait or emotional reactivity, all *ps* > 0.098. Modeling emotional reactivity through HR or HRV variables in the above analyses did not reveal significant interactions with the factors of interest.

## Discussion

This study addressed several core questions that have been debated in the emotional eating literature for decades and obscured its theoretical and clinical potential. To answer the question about who shows potentially problematic emotional overeating after experimental negative emotion induction, we contrasted emotional eating/emotion regulation theories with cognitive disinhibition/restrained eating theories by modeling trait restrained and emotional eating as predictors of appetitive food image responses. To answer the question of mechanisms, or the “how” of emotional overeating, a wide range of measures was taken, but particularly prefrontal brain activity allowed for contrasting these two models. Finally, the question of individual differences in emotional reactivity was assessed to uncover a possible dose-response relationship between emotion strength and emotional overeating (“fight or flight”). We based this study on Blechert et al. (2014) and sought to replicate findings and improve some design issues. First, we included neutral objects as an additional control to capture food-specific effects only. Second, we presented the same set of food pictures in both conditions. Third, we standardized starting time and lunch options. Last, we increased the sample size and variance in trait emotional and restrained eating. Results can be summarized as follows.

### Evidence for Emotional Overeating in Trait Emotional Eating: Increase in Experiential and Facial Appetitive Responding in Negative Emotional States

As expected under the emotion regulation theory, pleasantness ratings of food pictures (compared to object pictures) were fully moderated by trait emotional eating (and not by restrained eating). Trait emotional eaters rated foods as more pleasant in the negative experimental condition compared to the neutral experimental condition, whereas individuals with lower scores conversely rated food as less pleasant when in a negative emotional state. This pattern replicated our results in the 2014 study, where we found similar opposing patterns in high and low emotional eaters for food craving ratings. Further, it extended to findings of the facial corrugator muscle: More emotional eaters showed a more appetitive reaction – less “frowning” – to food images (relative to object images) in the negative relative to the neutral condition. The relationship of corrugator EMG with bipolar pleasantness is relatively well established (Larsen et al., 2003) lending high validity to this result. The consistency of subjective pleasantness ratings and objective corrugator responses indicates that results are not a mere reflection of social desirability or participants’ self-concepts of their reactivity to food during negative emotions. Adding to that, desire to eat ratings as a measure with a more direct relationship to eating behavior (Boswell and Kober, 2016) generally confirmed the pattern in palatability and EMG measures, i.e., the moderation of emotional overeating by trait emotional eating. Yet, only individuals with a robust emotional response to the idiosyncratic emotion induction showed a relative increase in desire to eat in the negative condition.

Concluding, it seems that the “normative response” to negative emotional states in people reacting strongly to the emotion induction is appetite suppression (strong reductions in desire to eat in the negative condition). Only in highly emotional eaters, this suppressive effect reversed. Thus, on desire to eat, emotional overeating in high trait emotional eating is only relative in nature, i.e., when contrasted against the strong appetite suppression in low emotional eating. Hence, trait emotional eating can be defined as not showing the normative stress response of decreased food intake but rather an equal or increased intake compared to other situations (Van Strien, 2010).

### Individual Differences in Emotional Reactivity: Rating and Neural Effects

Interestingly, in addition to interacting with emotional eating on desire to eat ratings, emotional reactivity also showed an independent effect: motivated attention to foods, as indexed by the P300, was increased under negative emotion in low responders, suggesting that their appetitive value counters mild negative affect (independent of emotional and restrained eating). This effect reversed in high responders: Their food-specific P300 decreased during negative emotions, maybe because immersive emotional recollection processes distracted from the foods or brain activity shifted to emotion-related or appetitive areas insufficiently measured in EEG. This resonates with physiological theories of emotional eating: low intensity emotional states seem to trigger compensatory appetitive attentional mechanisms, whereas high intensity emotional states (“fight or flight” mode) reverse this pattern. Thus, emotional reactivity should be seen as independent moderator of emotional overeating in addition to its interactive contribution to trait emotional eating, which has important methodological implications for future studies.

### Restrained Eating Modulates Indices of Attention, but Not Experience or Facial Expression

In contrast to the predictions of the disinhibition theory, trait restrained eating did not moderate condition effects on rating and EMG indices. This contradicts cognitive models suggesting that restrained eaters are sensitive to emotional overeating (Evers et al., 2018; Ruderman, 1985). On a neural level, most dual process models of restrained eating assume an engagement of effortful top-down (prefrontal) mechanisms in following a weight control goal (Stroebe et al., 2013). However, in our study only the parieto-occipital P300 amplitude, a putative index of attention to motivationally salient stimuli, varied as a function of trait restrained eating. Individuals high in restraint showed increased activity to foods (relative to objects) in the negative (relative to the neutral) condition, which would indicate that they become susceptible to the foods (Papies et al., 2008; Werthmann et al., 2015). Yet, no “downstream” effects on ratings or facial responses were seen, suggestive of “successful” preparatory appetite regulation. Thus, instead of effortful, prefrontal, top-down control, they might have used more efficient means of regulation e.g., recurrence on habitual food choice rules (Adriaanse et al., 2011a). In addition, other than in emotional eating, restrained eating correlations with P300 did not depend on emotional reactivity and thus might be unrelated to the strength of negative states. Trait restrained eating can thus be conceptualized as an independent mechanism in emotional overeating centered on attentional control.

### Limitations, Future Directions, and Conclusion

Our study included only women, mandating further research in men. Although disordered eating is more common among women, prevalence studies show that disordered eating in men most frequently expresses in binge eating without compensatory behavior and that compared to women, men are less likely to seek help (Kessler et al., 2013). Thus, the role of negative emotions on overeating and possible gender differences would add to the understanding of disordered eating in this understudied population. Additionally, food ratings on pleasantness and desire to eat represent a disposition to eat but remain inconsequential to the individual compared to real food intake. We intentionally opted against a taste-test to measure food intake after each condition because taste tests are consistently prone to effects of observation and social desirability considerations (Robinson et al., 2015) that cannot be excluded entirely. Furthermore, actual food intake can be modulated by self-regulatory processes such as meal planning, sensory specific satiety, diurnal food preferences, and effort-reward considerations (monetary value of foods), among others. Thus, we consider ratings (and to some degree EMG/EEG responses) as a propensity to consume respective foods upon availability and situational adequacy. However, the natural occurrence of emotional overeating in strong responders to the laboratory task might be followed up in daily life, e.g., by using EMA measures.

Although affective ratings (PANAS) confirmed a successful induction of negative emotion in the sample as a whole, 21.5% of the participants did not show the expected changes on PANAS ratings, i.e., they did not report more NA after the negative condition than after the neutral condition. Idiosyncratic scripts have been deemed as effective and used in patients (Cuthbert et al., 2003; Hilbert et al., 2011) and are consistent with capturing naturalistic stressors akin to EMA studies (Wilhelm and Grossman, 2010). However, they add variability compared to standardized emotion induction protocols such as affective picture or video exposure (Evers et al., 2010) and performance or social stressors (Van Strien et al., 2012) and might be less severe in sum. Effects of emotional reactivity did not go as far as altering autonomic arousal systems: other than in Hilbert et al. (2011) no condition effects were detected on cardiac activity, which might be due to the much longer and rather passive emotion induction.

As emotional overeating might be a subclinical form of binge eating, an important future direction would be to apply the present paradigm to individuals with eating disorders like Anorexia Nervosa or Bulimia Nervosa. These disorders are characterized by emotion regulation difficulties (Naumann et al., 2016) and show both patterns of either emotional overeating (binging) or undereating/restricting (Meule et al., 2019). Further, explicit emotion regulation instructions (Svaldi et al., 2014) could be added to the present task to narrow down the role of emotional overeating and identify more adaptive alternatives that effectively abate negative emotions and their “spill over” effects on food cues. Cognitive disinhibition models require further investigation, maybe by inducing “diet breaches” (i.e., prior consumption of forbidden foods) in combination with emotion inductions (Herman and Polivy, 1975; Ruderman, 1986). To further assess subgroup differences within restrained eaters, different types of restraint could be classified in terms of cognitive resources, dieting success, rigidity, and other pathological eating styles (Papies et al., 2008; Meule et al., 2012). Future research might follow up on our P300 effects with more sophisticated EEG analyses (e.g., source level connectivity analyses) to elaborate the role of frontal/regulatory and occipito-parietal/attentional cue reactivity networks in emotional and restrained eating.

In conclusion, we were able to characterize a multi-layered emotional eating signature in trait emotional eaters and restrained eaters. Support for an emotion-regulation framework was found in trait emotional eating and emotional reactivity was identified as a potential boundary condition for the effect to emerge. Although recent work emphasizes the importance of restraint and failure in self-regulation as a cause for emotional overeating (Evers et al., 2018) our findings are more in line with emotional eating theories of food reducing negative affect, a negatively reinforced strategy probably learnt early on in life (Snoek et al., 2007). Restrained eaters attribute more attentional resources to food in negative emotional states but lack corresponding appetitive responses. Our findings might aid future theorizing and research on factors predicting emotional overeating.

## Data Availability Statement

The raw data supporting the conclusions of this article will be made available by the authors, without undue reservation.

## Ethics Statement

The studies involving human participants were reviewed and approved by Ethics Committee of the University of Salzburg, Austria. Written informed consent to participate in this study was provided by the participant, or, if underage, by the participants’ legal guardian/next of kin.

## Author Contributions

RS conducted data preparation and statistical analysis and drafted the manuscript. CG helped to writing the study protocol and to collect and structure data. KE helped with the preparation and analysis of the EEG data. A-KA helped with the preparation and analysis of the EMG data. FW contributed to the final draft. CV designed the study, acquired funding, helped writing the study protocol and contributed to the final draft. AL helped writing the study protocol and contributed to the final draft. ZD helped writing the study protocol and contributed to the final draft. JB designed the study, acquired funding, helped writing the study protocol and contributed to the final draft.

## Conflict of Interest

The authors declare that the research was conducted in the absence of any commercial or financial relationships that could be construed as a potential conflict of interest.
