# Supplementary material for: Fight, Flight, – Or Grab a Bite! Trait Emotional and Restrained Eating Style Predicts Food Cue Responding Under Negative Emotions
Source: Front Behav Neurosci. 2020 Jun 3;14:91. doi: 10.3389/fnbeh.2020.00091 (PMC7283754; doi:10.3389/fnbeh.2020.00091)
Supplement: Supplementary file 1 [file Data_Sheet_1.docx]

Supplementary Material

**Supplement A**

*Supplementary Figure 1.* Scatterplot of DEBQ questionnaire scores of trait emotional and restrained eating.


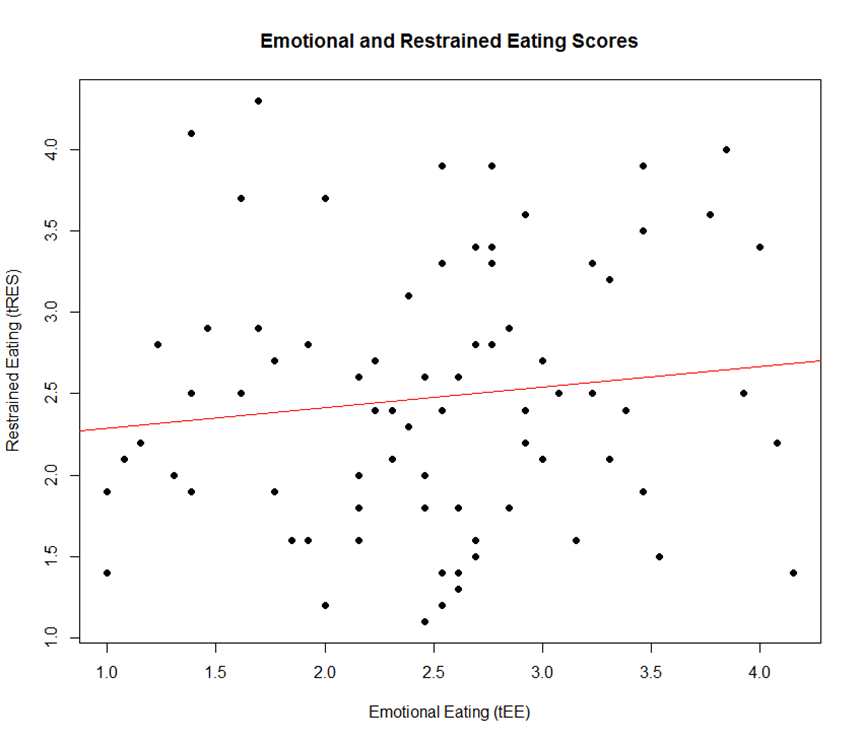


**Supplement B: Predictor values for restrained eating on pleasantness**

Supplementary Table 1

*Full Multilevel-Models of Pleasantness* *and Corrugator Electromyography for a food-object difference score with trait restrained eating as a predictor (number of observations: N=146).* *No reduction was applied, since there was no significant predictor.*

|  | **Full Model Pleasantness** | **Full Model Corrugator** |
| --- | --- | --- |
| **Fixed Effects** | *b* (SE) | *b* (SE) |
| (Intercept) | 22.1 (2.02)*** | -0.06 (0.04) |
| **Predictors** |  |  |
| Condition | 0.93 (1.3) | 0.05(0.05) |
| Condition Order | -0.72(2.89) | 0.06(0.06) |
| NA Baseline | -0.13(0.41) | 0.01(0.01) |
| NER | -0.17(0.33) | 0.008(0.008) |
| tRES | -0.32(2.02) | 0.08(0.05) |
| **Interactions** |  |  |
| Condition × NER | 0.07(0.27) | -0.001(0.01) |
| Condition × tRES | -0.04(0.16) | -0.09(0.06) |
| NER × tRES | -0.07 (0.04)^(*)^ | -0.01(0.01) |
| Condition × NER × tRES | 0.02(0.03) | 0.003(0.01) |
| **Random Effects** | Variance (*SD*) | Variance (*SD*) |
| Participant (Intercept) | 129(11.3) | 0.03(0.17) |
| Residual | 64.9(8.06) | 0.09(0.30) |
| AIC | 1248 | 164 |

Note: NER =negative emotional reactivity, tRES =trait restrained eating. Significance codes: ‘***’=*p*<001; ‘**’=*p*<0.01; ‘*’=*p*<0.05; ‘^(*)^’=*p*<0.1.

**Supplement C: Predictor values for restrained eating on desire to eat**

Supplementary Table 2

*Full Multilevel-Models of desire to eat rating for food only, modeled on trial level with trait restrained eating as a predictor (number of observations: N=4108).*

|  | **Full Model DTE** | **Reduced Model DTE** |
| --- | --- | --- |
| **Fixed Effects** | *b* (SE) | *b* (SE) |
| (Intercept) | 52.23(3.34)*** | 54.4(2.62)*** |
| **Predictors** |  |  |
| Condition | 5.55(1.82)** | 5.59(1.78)** |
| Trial | 0.02(0.02) |  |
| Condition Order | 1.50(3.41) |  |
| NA Baseline | 0.31(0.48) |  |
| NER | -0.31(0.39) |  |
| tRES | -1.56(2.37) |  |
| **Interactions** |  |  |
| Condition × NER | 0.58(0.37) |  |
| Condition × tRES | 1.44(2.25) |  |
| NER × tRES | -0.68(0.48) |  |
| Condition × NER × tRES | 0.21(0.44) |  |
| **Random Effects** | Variance (*SD*) | Variance (*SD*) |
| Participant (Intercept) | 232(15.2) | 226(15.0) |
| Participant \| Condition | 178(13.3) | 179(13.4) |
| Picture Number (Intercept) | 91.9(9.59) | 91.9(9.59) |
| Residual | 927(30.4) | 927(30.4) |
| AIC | 40112 | 40102 |
| χ² | .566 | |

Note: DTE =desire to eat, NER =negative emotional reactivity, tRES =trait restrained eating. Adding a random slope for Participant significantly improved DTE predictions (*p*<.001). Significance codes: ‘***’=*p*<001; ‘**’=*p*<0.01; ‘*’=*p*<0.05; ‘.’=*p*<0.1.

**Supplement D: Predictor values for P300**

Supplementary Table 3

*Full and reduced Multilevel-Models of P300 activity for a food-object difference score with trait restrained eating as a predictor.*

|  | **Full Model P300** | **Reduced Model P300** |
| --- | --- | --- |
| **Fixed Effects** | *b* (SE) | *b* (SE) |
| (Intercept) | -0.28 (0.14)* | -0.30 (0.09)*** |
| **Predictors** |  |  |
| Condition | 0.10 (0.15) |  |
| Condition Order | -0.12(0.18) |  |
| NA Baseline | 0.04(0.03) |  |
| NER | -0.05(0.02)* | -0.05 (0.02)* |
| tRES | 0.26(0.14) ^(*)^ | 0.29(0.14)* |
| **Interactions** |  |  |
| Condition × NER | 0.08(0.03)** | 0.09(0.03)** |
| Condition × tRES | -0.37(0.18)* | -0.37(0.18)* |
| NER × tRES | -0.007 (0.03) |  |
| Condition × NER × tRES | 0.01(0.03) |  |
| **Random Effects** | Variance (*SD*) | Variance (*SD*) |
| Participant (Intercept) | 0.15(0.39) | 0.16(0.40) |
| Residual | 0.73(0.86) | 0.72(0.85) |
| AIC | 386 | 380 |

| χ² | .645 |
| --- | --- |

Note: NER =negative emotional reactivity, tRES =restrained Eating. Significance codes: ‘***’=*p*<001; ‘**’=*p*<0.01; ‘*’=*p*<0.05; ‘^(*)^’=*p*<0.1.

**Supplement E: Frontal EEG**

Following our a-priori prediction of frontal effects based on our previous findings (Blechert et al., 2014), a broad bilateral frontal ERP cluster in the LPP time range (1LB, 1RB, 2LB, 2RB, 2L, 2R, 1Z, 2Z) and a right-frontal cluster (1R, 1RB, 2RB, 2RC, 1RD, as in previous report) between 300-600ms were extracted. However, a right frontal effect in the LPP time range was not visible upon inspection of difference waves. Testing for potential higher order interactions revealed a significant Emotional Eating×Emotional Reactivity interaction, *b*=0.09 (SE=0.03), *p*=.003, and a Condition×Emotional Eating× Emotional Reactivity interaction, *b*=-0.08 (SE=0.04), *p*=.041 for the bilateral cluster. Among high-reacting individuals, high emotional eaters showed increased, widespread, positive going LPP amplitudes in the negative relative to the neutral condition. Emotionally responsive individuals high in emotional eating might seek engagement with appetitive foods to instrumentally down-regulate negative emotional states. By contrast, no significant main effects or interactions with trait restrained eating as a predictor were found, all *p*s>.072.


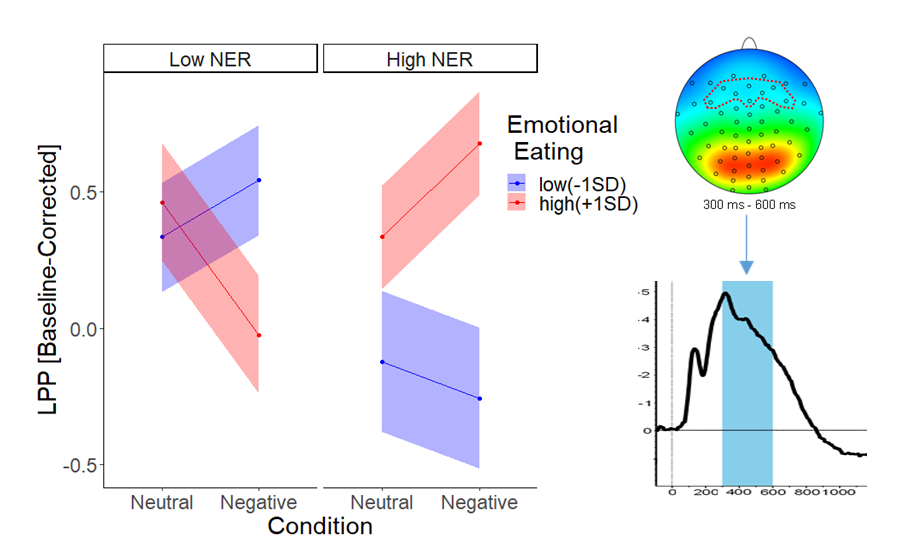


*Supplementary Figure 2.* ERP in the LPP time range (300-600ms; blue bar in waveform graph) at a broad frontal bilateral cluster (area circled with dotted line) for high and low trait emotional eating as well as high and low NER (+-1 SD).

**
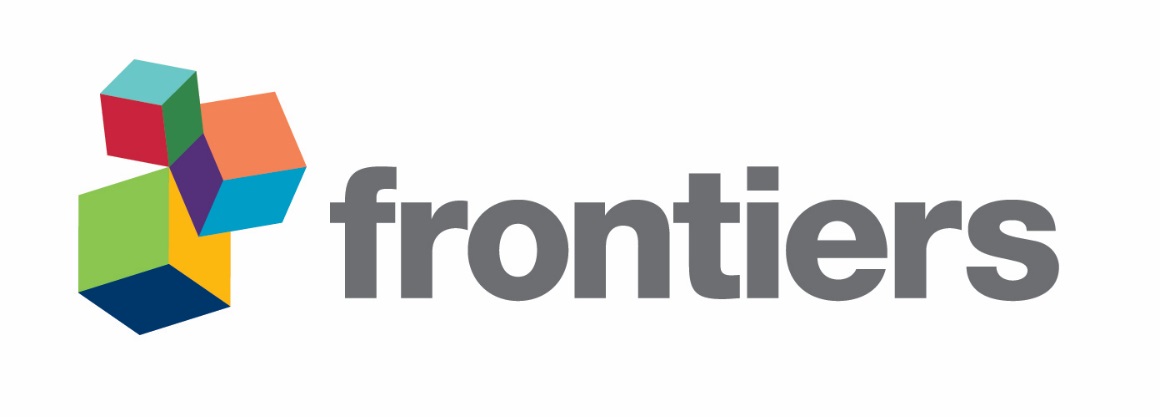
**
